# UV-Schutz-Bündnis in Deutschland – Zweck und Ziele

**DOI:** 10.1007/s00347-021-01543-w

**Published:** 2022-01-17

**Authors:** Cornelia Baldermann, UV-Schutz-Bündnis, Carola Berking, Claas Ulrich, Eckhard Breitbart, Henriette Bunde, Beate Volkmer, Winfried Janßen, Cornelia Baldermann, Daniela Weiskopf, Frauke Grimm, Birgit Keller, Vinodh Kakkassery, Ralph von Kiedrowski, Thomas Stavermann, Sophia Schlette, Tanja-Maria Kessel, Mark Berneburg, Peter Höger, Manigé Fartasch, Claudine Strehl, Ernst Engelmayr, Ute Mons, Dirk Schadendorf, Ulrike Helbig, Ludwig M. Heindl, Gudrun Laschewski, Eggert Stockfleth, Rüdiger Greinert, Joachim Hübner, Alexander Katalinic, Peter Mohr, Birgit Pieper, Martin Brose, Wolfgang Panter, Helmut Schlager, Dirk Schäfermeyer

**Affiliations:** 1 Koordinierungsstelle UV-Schutz-Bündnis am Bundesamt für Strahlenschutz, Bundesamt für Strahlenschutz, Neuherberg, Deutschland; 2 Koordinierungsstelle UV-Schutz-Bündnis am Bundesamt für Strahlenschutz, Fachgebiet WR4 – Optische Strahlung, Bundesamt für Strahlenschutz, Ingolstädter Landstr. 1, 85764 Neuherberg, Deutschland

**Keywords:** UV-Strahlung, Gesundheitsrisiken, Prävention, Maßnahmen, Konsolidierung, UV radiation, Health risks, Prevention, Measures, Consolidation

## Abstract

Trotz der ernsten gesundheitlichen Gefahren von UV-Strahlung ist bis heute der Schutz vor UV-Strahlung keine Selbstverständlichkeit. Die Einschätzung der Gesundheitsrisiken durch UV-Strahlung in der Bevölkerung ist zwar weitestgehend realistisch, scheint aber nicht zu einer veränderten Einschätzung des persönlichen Risikos und zu einem adäquaten UV-Schutz-Verhalten zu führen. Hierzu tragen nicht zuletzt die teils widersprüchlichen Aussagen und Empfehlungen bezüglich der positiven und negativen Gesundheitsfolgen – auch aus Wissenschaftskreisen – bei. Eine Harmonisierung sowie eine Bündelung der Aussagen und Aktivitäten einzelner Akteure im UV-Schutz zur Prävention UV-bedingter Erkrankungen verleiht den Schlüsselbotschaften das notwendige Gewicht, um UV-Schutz zu einer Selbstverständlichkeit in der Gesellschaft werden zu lassen. Zu diesem Zweck wurde das UV-Schutz-Bündnis vom Bundesamt für Strahlenschutz (BfS) initiiert. In diesem Beitrag wird über das UV-Schutz-Bündnis berichtet, es werden die Partner im Bündnis, die Ziele des UV-Schutz-Bündnisses, bisherige Arbeitsergebnisse des Bündnisses sowie Aktionen und Interventionen der Bündnispartner vorgestellt. Die Wirkung des UV-Schutz-Bündnisses nach außen wird beschrieben und ein Ausblick auf die weiteren Aufgaben gegeben, denen sich das Bündnis gegenübergestellt sieht.

## Abstract (en)

Despite the serious health consequences of UV radiation, protection against UV radiation is even now still not a matter of course. The population has a largely realistic view of UV radiation-related health risks but this does not seem to instigate a change in personal risk assessments and towards an adequate UV protection behavior. This is not least due to the partly contradictory statements and recommendations regarding the positive and negative health effects, also from the scientific community. A harmonization as well as a collation of the statements and activities of individual players in UV protection related to the prevention of UV-related diseases gives the key messages the necessary strength to make UV protection a matter of course in society. To this end, the UV Protection Alliance was initiated by the Federal Office for Radiation Protection (BfS). This article reports on the UV Protection Alliance, presents the partners in the Alliance, the goals of the UV Protection Alliance, previous results of work by the Alliance and actions and interventions of the Alliance partners. The public impact of the UV Protection Alliance is explained and an outlook is given on future tasks of the Alliance.

Mit der Sonnenstrahlung erreicht optische Strahlung – Infrarotstrahlung, sichtbares Licht und ultraviolette (UV) Strahlung – die Erdoberfläche. Sonnenstrahlung wird allgemein als sehr positiv wahrgenommen – v. a. wegen der Helligkeit und der Wärme, wodurch unter anderem die Gesundheit, das Wohlgefühl und damit auch die Psyche gestärkt werden. Der energiereichste Teil der optischen Strahlung, die UV-Strahlung, umfasst den Wellenlängenbereich von 100–400 nm, wobei aufgrund der filternden Wirkung der Erdatmosphäre und vornehmlich der stratosphärischen Ozonschicht nur UV-Strahlung der Wellenlängen von ca. 290–400 nm die Erdoberfläche erreicht. UV-Strahlung ist für den Menschen nicht wahrnehmbar und bereitet ernste gesundheitliche Probleme – egal, ob es sich dabei um natürliche UV-Strahlung der Sonne oder um künstlich erzeugte UV-Strahlung beispielsweise in Solarien handelt. 

UV-Strahlung ist für den Menschen nicht wahrnehmbar und bereitet ernste gesundheitliche Probleme

Die einzig aktuell bekannte positive Wirkung der UV-Strahlung liegt darin, dass ein Teil der UV-Strahlung, die UV-B-Strahlung mit Wellenlängen von 280–315 nm, die Synthese des körpereigenen Vitamin D anstößt. Gleichzeitig verursachen diese UV-B-Strahlung und UV-Strahlung insgesamt zahlreiche akut und später im Leben auftretende Gesundheitsschäden an Augen und Haut. Am Auge können beispielsweise akut Bindehaut- oder Hornhautentzündungen auftreten. Langfristig trägt UV-Strahlung unter anderem zur Kataraktbildung [1] und zu Krebserkrankungen an und im Auge bei [2, 3]. Bei degenerativen Netzhauterkrankungen wie dem Morbus Stargardt und der altersabhängigen Makuladegeneration konnte eine Beteiligung von UV-Strahlung bisher nicht vollständig ausgeschlossen werden [4]. Die bekanntesten Folgen einer übermäßigen UV-Bestrahlung für die Haut sind Sonnenbrände, Sonnenallergien, vorzeitige Hautalterung mit übermäßiger Faltenbildung und im schlimmsten Fall Hautkrebs. Ursache für die Krebserkrankungen ist, dass UV-Strahlung direkt (UV-B- und UV-C-Strahlung^1^) und indirekt (UV-A-Strahlung) das Erbgut schädigt und kanzerogen ist [5]. Natürliche wie künstlich erzeugte UV-Strahlung ist darum von der Internationalen Agentur für Krebsforschung (International Agency for Research on Cancer [IARC]) der höchsten Risikogruppe I krebserregender Agenzien als „krebserregend für den Menschen“ zugeordnet [6]. UV-Strahlung ist Hauptursache für Hautkrebs, an dem in Deutschland jährlich rund 4000 Menschen versterben – im Jahr 2019 waren es 4097 Personen [7]. Laut dem BARMER GEK Arztreport von 2014 waren 2012 in Deutschland knapp 1,6 Mio. Menschen von einer Hautkrebsdiagnose betroffen [8]. Zwischen den Jahren 2011 und 2018 erhöhten sich für den hellen Hautkrebs die Betroffenenzahlen um 35 % von rund 1,23 Mio. auf 1,66 Mio., beim kutanen malignen Melanom, dem schwarzen Hautkrebs, um 24,7 % von rund 300.000 auf 374.000 Personen [9].

Trotz dieser ernsten gesundheitlichen Konsequenz der UV-Strahlung ist bis heute der Schutz vor UV-Strahlung keine Selbstverständlichkeit. Ein informierter und ausgewogener Umgang mit UV-Strahlung tut darum Not. Umfragen zufolge ist die Einschätzung der Gesundheitsrisiken durch UV-Belastung (UV-Exposition) in der Bevölkerung zwar realistisch [10], scheint aber nicht unbedingt zu einer veränderten Einschätzung des persönlichen Risikos und zu einem adäquaten UV-Schutz-Verhalten zu führen. Hierzu tragen nicht zuletzt die teils widersprüchlichen Aussagen und Empfehlungen bezüglich der positiven und negativen Gesundheitsfolgen – auch aus Wissenschaftskreisen – bei. Warnungen und Informationen scheinen nur bedingt wahrgenommen zu werden. Den Schlüsselbotschaften ist darum mehr Gewicht zu verleihen. Ein Weg hierfür ist, einzelne Stimmen zu bündeln, d. h. in diesem Zusammenhang die einzelnen Akteure in den Bereichen „Gesundheit und UV-Schutz“ sowie „Prävention UV-bedingter Erkrankungen“ zu vernetzen und als Netzwerk gemeinsam zu agieren. Das Bundesamt für Strahlenschutz (BfS) hat darum 2011 das UV-Schutz-Bündnis initiiert.

## UV-Schutz-Bündnis

Das BfS ist eine selbstständige wissenschaftlich-technische Bundesoberbehörde im Geschäftsbereich des Bundesministeriums für Umwelt, Naturschutz, nukleare Sicherheit und Verbraucherschutz (BMUV) und arbeitet für die Sicherheit und den Schutz der Menschen und der Umwelt vor Schäden durch ionisierende und nicht ionisierende Strahlung – und somit auch für den Schutz vor den Schäden der UV-Strahlung. Hierfür pflegt das BfS seit seiner Gründung 1989 einen regen Austausch mit dermatologischen Fachgesellschaften. Das Thema betrifft aber zahlreiche andere Behörden und medizinische Fachrichtungen in Deutschland und Europa sowie Institutionen, die aktiv den UV-Schutz und die Prävention UV-bedingter Erkrankungen – und nicht nur der Haut, sondern auch der Augen – fördern und unterstützen. Es fehlte demnach eine Art Netzwerk aller im UV-Schutz engagierten Behörden, Arbeitsgemeinschaften, Fachgesellschaften, gemeinnützigen Organisationen, Institute, Stiftungen, Verbände und Vereine aus den Bereichen Wissenschaft, Strahlenschutz, Arbeitsschutz, Medizin, Prävention, Gesundheitsförderung und Gesundheitsversorgung. Zusätzlich stellt die Vernetzung der Hauptakteure im Bereich UV-Schutz einen wichtigen Schritt dar, um die Risikowahrnehmung in der Bevölkerung zu verbessern. Denn gemeinsam getragene, gleichlautende Aussagen und Empfehlungen aus dem Mund zahlreicher unterschiedlicher Akteure bewirken mehr, als wenn dieselbe Nachricht in unterschiedlichem Wortlaut durch Einzelne verkündet wird. Im Jahr 2011 startete darum das BfS eine Initiative zur Kooperation, zunächst mit 13 teilnehmenden Institutionen. Im Jahr 2012 wurde mit Unterzeichnung der „Vereinbarung zur Zusammenarbeit“ [11] durch diese 13 Partner das UV-Schutz-Bündnis gegründet. Mittlerweile ist das Bündnis auf insgesamt 27 Partner angewachsen (Tab. 1). Koordiniert wird das UV-Schutz-Bündnis vom BfS. Basis der Arbeit des UV-Schutz-Bündnisses sind wissenschaftliche Forschungsergebnisse, die nach erfolgreichem Begutachtungsverfahren („peer review“) in wissenschaftlichen Fachzeitschriften veröffentlicht wurden, sowie Übersichtsartikel und Berichte, die diese Forschungsergebnisse zusammenfassend betrachten und bewerten.

**Table 1:** 

Bündnispartner	Beitrittsjahr
*Arbeitsgemeinschaft Dermatologische Onkologie (ADO)*	2013
*Arbeitsgemeinschaft Dermatologische Prävention e.* *V. (ADP)*	2011
*Berufsverband der Augenärzte Deutschlands e.* *V. (BVA)*	2021
*Berufsverband der Deutschen Dermatologen e.* *V. (BVDD)*	2011
*Bundesamt für Strahlenschutz (BfS)*	2011
*Bundesanstalt für Arbeitsschutz und Arbeitsmedizin (BAuA)*	2013
*Bundesministerium für Umwelt, Naturschutz, nukleare Sicherheit und Verbraucherschutz (BMUV)*	2021
*Bundeszentrale für gesundheitliche Aufklärung (BZgA)*	2011
*Deutsche Dermatologische Gesellschaft (DDG)*	2011
*Deutsche Gesellschaft für Kinder- und Jugendmedizin e.* *V. (DGKJ)*	2011
*Deutsche Gesetzliche Unfallversicherung e.* *V. (DGUV) vertreten durch:*	2015
– Institut für Arbeitsschutz der DGUV (IFA)	2015
– Institut für Prävention und Arbeitsmedizin der DGUV, Institut der Ruhr-Universität Bochum (IPA)	2015
– Sachgebiet Hautschutz im DGUV Fachbereich PSA (SG Hautschutz)	2018
– Sachgebiet Nichtionisierende Strahlung im DGUV Fachbereich ETEM (SG NIR)	2018
*Deutsche Krebsgesellschaft (DKG)*	2011
*Deutsche Ophthalmologische Gesellschaft (DOG)*	2016
*Deutscher Hausärzteverband e.* *V.*	2011
*Deutscher Wetterdienst (DWD)*	2013
*Deutsches Krebsforschungszentrum (DKFZ)*	2011
*European Skin Cancer Foundation (ESCF)*	2011
*European Society of Skin Cancer Prevention (EUROSKIN)*	2011
*Gesellschaft der epidemiologischen Krebsregister in Deutschland e.* *V. (GEKID)*	2011
*Nationale Versorgungskonferenz Hautkrebs e.* *V. (NVKH)*	2021
*Stiftung Deutsche Krebshilfe*	2011
*Verband deutscher Betriebs- und Werksärzte e.* *V. (VDBW)*	2011
*Wissenschaftliches Institut für Prävention im Gesundheitswesen der Bayerischen Landesapothekerkammer (WIPIG)*	2019
*Zentralverband der Augenoptiker und Optometristen (ZVA)*	2017

## Ziele des UV-Schutz-Bündnisses

Übergeordnetes Ziel ist die Steigerung der Lebensqualität in Deutschland durch Reduzierung übermäßiger UV-Belastung mittels flächendeckender Anwendung verhältnispräventiver Maßnahmen im Einklang mit verhaltenspräventiven Maßnahmen [12] an Orten, an denen sich Menschen diesem Umweltfaktor in ihren Lebenswelten^2^ vermehrt aussetzen^3^ oder ihm ausgesetzt sind. Dieses Ziel steht im Einklang mit internationalen und nationalen Empfehlungen zur Prävention von Hautkrebs [5, 13, 14] sowie mit den Vorgaben der Nationalen Dekade gegen Krebs [15] und dem Handlungsfeld 1 „Prävention von Hautkrebs“ [16] und dem Versorgungsziel „Ziel 1: Die Bevölkerung wird vor der Entstehung von Hautkrebs wirksam geschützt“ [17] der nationalen Versorgungskonferenz Hautkrebs (NVKH) e. V. [18].

Ziel ist es, langfristig die Zahl UV-bedingter Erkrankungen zu reduzieren

Die Bündnispartner treten gemeinsam für einen verantwortlichen Umgang mit der Sonne und für einen gelebten UV-Schutz ein. Ziel ist es, langfristig die Zahl UV-bedingter Erkrankungen, insbesondere Krebserkrankungen, zu reduzieren. Das Bündnis fordert und fördert verhaltens- und verhältnispräventive Maßnahmen, d. h. die Etablierung und Anwendung der richtigen Maßnahmen und der passenden Strukturen in allen Lebenswelten, die geeignet sind, UV-bedingten Erkrankungen, besonders Krebserkrankungen, vorzubeugen. Das UV-Schutz-Bündnis versteht UV-Schutz als gesamtgesellschaftliche Aufgabe. In enger Kooperation der Partner wird der wissenschaftliche Kenntnisstand zu positiven und negativen gesundheitlichen Wirkungen der UV-Strahlung sowie Verhaltens- und Verhältnisprävention aufgearbeitet und in verständliche Worte gefasst. Im Rahmen von Fachgesprächen werden einzelne Themen aufgegriffen und zusammen mit hierfür geladenen Expert*innen offene Aspekte geklärt. Basierend auf dem wissenschaftlichen Kenntnisstand, werden Schutzkonzepte und konsentierte Empfehlungen entwickelt. Das Bündnis engagiert sich für die politische Verankerung des Themas „Prävention UV-bedingter Erkrankungen durch UV-Schutz“ und fokussiert v. a. die Etablierung verhältnispräventiver Maßnahmen. Alle Partner arbeiten auf das Ziel hin, UV-Schutz erlern- und erlebbar zu machen.

## Aktionen/Interventionen der Bündnispartner

Die verschiedenen Bündnispartner sind mit Projekten und Forschung in vielen Bereichen des Alltags aktiv, um einen verantwortungsvollen Umgang mit UV-Strahlung in der Praxis zu fördern und zu stärken. Im Folgenden wird eine kleine Auswahl vorgestellt.

### Projekte in den Lebenswelten von Kindern und Jugendlichen

**Figure 1:**
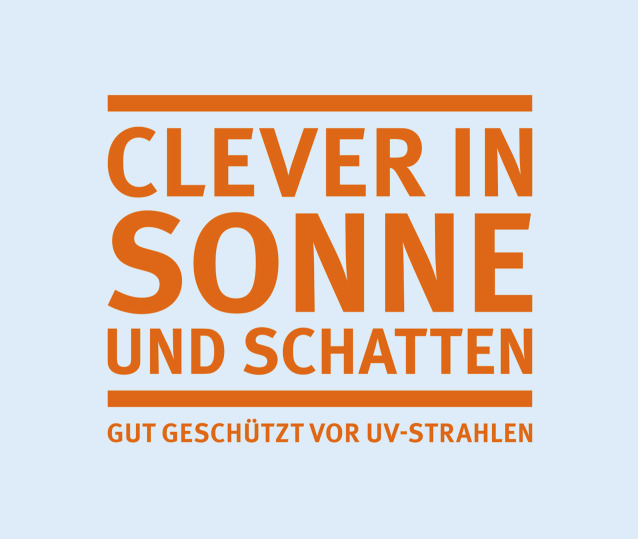

Da Kinderhaut gegenüber der UV-Strahlung der Sonne besonders empfindlich reagiert, ist Sonnenschutz von klein auf dringend geboten. Entsprechende Interventionen in den Lebenswelten von Kindern und Jugendlichen werden von etlichen Bündnispartnern umgesetzt. Als gute Praxisbeispiele können genannt werden:

1. Clever in Sonne und Schatten
  Das von der Deutschen Krebshilfe geförderte Gemeinschaftsprojekt „CLEVER IN SONNE UND SCHATTEN“ der Deutschen Krebshilfe, der Uniklinik und der Universität zu Köln, des Nationalen Centrums für Tumorerkrankungen Dresden (NCT/UCC) und der Arbeitsgemeinschaft Dermatologische Prävention (ADP) e. V. (Logo s. Abb. 1) bringt UV-Schutz in die Lebenswelten von Kindern und Jugendlichen. Dazu gehören insbesondere Kitas, Grundschulen sowie Sport- und Freizeiteinrichtungen. Die zielgruppenspezifisch erstellten Projektmaterialien unterstützen Erzieher*innen, Lehrpersonen und Betreuungspersonal darin, Kinder altersgerecht zu richtigem Sonnenschutzverhalten anzuleiten und dazu zu motivieren, UV-Schutz in Theorie und Praxis selbstständig zu erlernen und anzuwenden. Die Materialien können kostenfrei bezogen werden. Einrichtungen können sich zudem für ihr UV-Schutz-Engagement als „Clever in Sonne und Schatten“ auszeichnen lassen [19].
2. SunPass – Gesunder Sonnenspaß für Kinder
  Die European Skin Cancer Foundation (ESCF) hat im Februar 2009 das Projekt „SunPass – Gesunder Sonnenspaß für Kinder“ zur Auszeichnung von Kindergärten für aktive Bemühungen im Sonnenschutz ins Leben gerufen. Seit 2012 beteiligen sich die Landeskrebsgesellschaften an diesem Projekt. Im Rahmen des Projekts werden gemeinsam Schutzmaßnahmen entwickelt, Erziehungskräfte und Eltern zum Sonnenschutz geschult und den Kindern spielerisch der Umgang mit der Sonne beigebracht [20].

### UV-Strahlungsbelastung verschiedener Berufsgruppen

**Figure 2:**
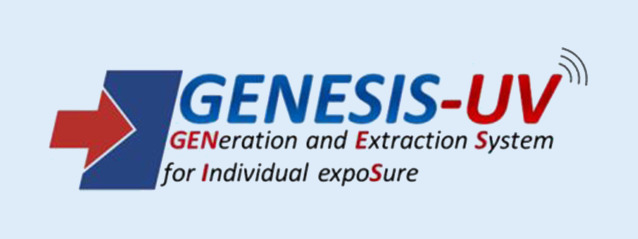

Die UV-Strahlungsbelastung von im Freien Beschäftigten wurde in der Vergangenheit deutlich unterschätzt. Wissenschaftliche Studien konnten eindeutig zeigen, dass Arbeitsnehmer*innen mit Außenbeschäftigung einem hohen Hautkrebsrisiko ausgesetzt sind [21]. Seit 2015 sind das Plattenepithelkarzinom und seine Vorstufen, die aktinischen Keratosen, als Berufskrankheit eingestuft [22, 23]. Derzeit mehren sich die Hinweise, dass Arbeitsnehmer*innen mit Außentätigkeiten auch ein erhöhtes Risiko für Basalzellkarzinome haben [24]. Für die Zukunft kann von deutlich steigenden Zahlen beruflich bedingter Hautkrebserkrankungen ausgegangen werden. Wie gefährdet verschiedene Berufsgruppen in dieser Hinsicht konkret sind, lässt sich nur mit tätigkeitsbezogenen Expositionsdaten abschätzen. Hierfür führt das Institut für Arbeitsschutz der Deutschen Gesetzlichen Unfallversicherung (IFA) im Rahmen des Projekts „Genesis-UV“ UV-Expositionsmessungen bei Beschäftigten im Freien und – neu – der UV-Belastung in der Freizeit durch (Logo s. Abb. 2; [25]).

### Aufklärung über die Gefahr von Solarien

**Figure 3:**
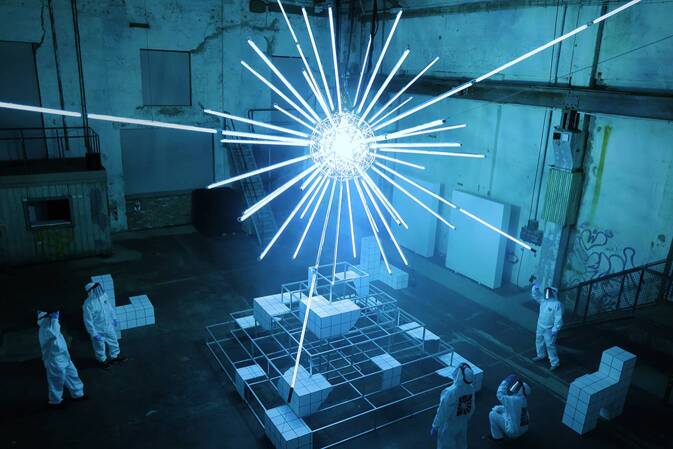

Solarien stellen eine aus wissenschaftlicher und medizinscher Sicht zur natürlichen UV-Strahlung unnötige zusätzliche UV-Strahlungsbelastung dar, die das Hautkrebsrisiko nur zusätzlich steigert [26]. Das BMUV, das BfS, die ADP (Arbeitsgemeinschaft Dermatologische Prävention e. V.), die Deutsche Krebshilfe und EUROSKIN engagieren sich auf wissenschaftlicher und politischer Ebene für nationale und europäische Regelungen bezüglich Solarien. Die Deutsche Krebshilfe und die ADP haben das Interventionsprogramm Solarien aufgelegt, das Anfang 2020 mit der Ausstellung eines spektakulären Kunstobjekts „Spectrum. The most dangerous artwork“ (Abb. 3) und einer hochkarätig besetzten Podiumsdiskussion für ein starkes Presseecho sorgte [27]. Das Kunstobjekt wurde 2020 im wichtigsten deutschen Kreativ-Wettbewerb durch den Art Director Club (ADC) unter anderem in den Kategorien Raumdesign, Licht und Event-Erfahrung 1‑mal mit Gold, 2‑mal mit Silber und 4‑mal mit Bronze ausgezeichnet. Weitere Auszeichnungen gewann die Installation in der Best of Content Marketing(BCM)-Preisverleihung.

### Wissenschaft und Forschung

Basis aller Aktivitäten ist der internationale wissenschaftliche Kenntnisstand. Auch die Bündnispartner führen Monitoring- und Forschungsprojekte durch oder fördern diese, wie z. B. die Deutsche Krebshilfe [28], die Deutsche Dermatologische Gesellschaft [29], die Nationale Versorgungskonferenz Hautkrebs (NVKH) e. V. [30] oder das Bundesamt für Strahlenschutz [31]. Die vom BfS aktuell initiierten Forschungsvorhaben im Bereich UV-Strahlung und Strahlenschutz beschäftigen sich mit der Entwicklung einer Geoinformationssystem(GIS)-basierten Software für Architekten zur Visualisierung der UV-Belastung in Abhängigkeit der Strukturen im Raum und mit der Erhebung etablierter verhältnispräventiver Maßnahmen wie schattengebende Strukturen in den Außenbereichen von Kindergärten und Schulen. Darüber hinaus misst das BfS im Rahmen des UV-Messnetzes [32] die den Erdboden erreichende solare UV-Strahlung und veröffentlicht den daraus abgeleiteten UV-Index – auch als Prognose [33]. Der Deutsche Wetterdienst (DWD) veröffentlicht auf Basis von Satellitendaten modellierte Prognosen des UV-Index [34]. Die Klinische Kooperationseinheit für Dermato-Onkologie des Deutschen Krebsforschungszentrums betreibt Forschung zu den Themen Vorbeugung, Diagnose und Therapie von Hauttumoren mit Fokus unter anderem auf Stammzelleigenschaften von Melanomzellen und Erforschung von neuen Biomarkern beim malignen Melanom. Etliche Bündnispartner richten darüber hinaus wissenschaftlich orientierte nationale wie internationale Tagungen und Konferenzen aus (z. B. [35–38]).

## Bisherige Arbeitsergebnisse des Bündnisses

**Figure 4:**
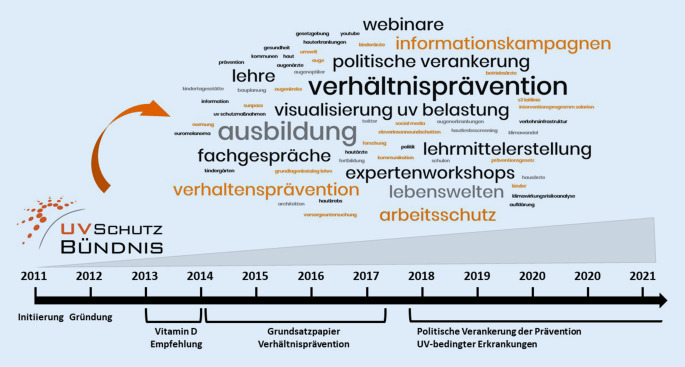

Seit der Initiierung des Bündnisses im Jahr 2011 bis heute wuchs das Bündnis von 13 auf derzeit 27 Partner an, veröffentlichte richtungsweisende Publikationen und widmete sich zunehmend intensiv unterschiedlichen Themen und der Umsetzung nachhaltiger Maßnahmen (Abb. 4).

Im Jahr 2011 wurde von den 13 Partnern des Bündnisses ein gemeinsames Faltblatt mit den wichtigsten Botschaften und Empfehlungen bezüglich des eigenen Sonnenschutzverhaltens herausgegeben (online abrufbar bis 2013). Neu daran waren nicht die Botschaften selbst, sondern dass diese Botschaften von 13 Institutionen ausgesprochen wurden, die zuvor nur zeitweilige themenbezogene Berührungspunkte zueinander hatten.

Im Jahr 2012 begann das UV-Schutz-Bündnis mit den Vorbereitungen zu fachübergreifenden Expertendiskussionen und einem interdisziplinären Fachgespräch bezüglich der notwendigen UV-Exposition zur Bildung des körpereigenen Vitamin D. An dem Fachgespräch im Jahr 2013 nahmen 20 namhafte Institutionen teil, neben den damaligen UV-Schutz-Bündnispartnern das Bundesinstitut für Risikobewertung (BfR), die Deutsche Gesellschaft für Ernährung e. V. (DGE), das Max-Rubner-Institut (MRI, Bundesforschungsinstitut für Ernährung und Lebensmittel) und das Robert Koch-Institut (RKI). Der fachübergreifende wissenschaftliche Diskurs hatte das Ziel, die widersprüchlichen Empfehlungen bezüglich der UV-Exposition zur Bildung des körpereigenen Vitamin D auf Basis des wissenschaftlichen Kenntnisstandes zu harmonisieren. Dies gelang – die konsentierte Empfehlung zur „UV-Exposition zur Bildung des körpereigenen Vitamin D“ ist seit Mitte 2014 auf den Internetseiten des BfS veröffentlicht und trug seither zur Versachlichung der Diskussion bei [39]. Im Jahr 2021 wurde die Empfehlung in die S3-Leitlinie „Prävention von Hautkrebs“ integriert und als konsentiertes Statement bestätigt [40].

Ende 2014 beschloss das UV-Schutz-Bündnis, ein Grundsatzpapier zur „Vorbeugung gesundheitlicher Schäden durch die Sonne – Verhältnisprävention in der Stadt und auf dem Land“ zu erarbeiten. Ausgangspunkt für diesen Beschluss war, dass die unvermindert steigenden Hautkrebsneuerkrankungszahlen darauf hindeuten, dass die seit Jahrzehnten propagierten verhaltenspräventiven Maßnahmen zu kurz greifen und um verhältnispräventive Maßnahmen zu ergänzen sind, also um Maßnahmen, durch die die Lebens- und Arbeitsumwelt derart gestaltet wird, dass ein rechtzeitiger Schutz vor übermäßiger UV-Belastung durch äußere Bedingungen wie Beschattung, Arbeitsprozessoptimierung und Anzeige der aktuellen UV-Belastung (UV-Index) ermöglicht wird [41] – dies v. a. auch vor dem Hintergrund, dass die Situation sich infolge des Klimawandels noch verschärfen kann [42, 43]. Auch die Problematik hoher UV-Strahlungsbelastung für das Auge konnte wissenschaftlich basiert integriert werden, nachdem 2016 die Deutsche Ophthalmologische Gesellschaft als Partner gewonnen werden konnte. Die Erarbeitung des Grundsatzpapiers nahm einen Zeitraum von über 2 Jahren ein. Im Jahr 2017 konnte das Papier veröffentlicht werden [44, 45].

Das Grundsatzpapier dient dem Ziel, im Freien, in Außenanlagen öffentlicher Einrichtungen sowie in den unterschiedlichen Lebenswelten der Bevölkerung verhältnispräventive Maßnahmen zum Schutz vor übermäßiger UV-Belastung und vor weiteren, durch den Klimawandel zunehmenden gesundheitsschädigenden Belastungen der Sonne (z. B. Hitzebelastung) flächendeckend zu etablieren. Die Maßnahmenentwicklung und -etablierung soll dabei unter Berücksichtigung der notwendigen Synergien von Verhaltens- und Verhältnisprävention und des Aspekts der Umweltgerechtigkeit erfolgen. Das UV-Schutz-Bündnis wendet sich mit diesem Papier unter anderem an Behörden, Sozialversicherungsträger und Sozialpartner, an Träger öffentlicher Einrichtungen, ausbildende oder ausbildungskoordinierende Organisationen sowie an die Medien. Es soll in einem gemeinsamen, kooperierenden Miteinander und unter Zuhilfenahme der den einzelnen Adressaten zur Verfügung stehenden Mittel und Möglichkeiten erreicht werden, dass Bürgerinnen und Bürger jeder Altersklasse Schutz vor übermäßiger UV-Belastung und – soweit dies mit UV-minimierenden Maßnahmen möglich ist – auch vor übermäßiger Hitzebelastung im Freien finden.

## Wirkung des UV-Schutz-Bündnisses nach außen

Etliche UV-Schutz-Bündnispartner setzen sich bereits seit Jahrzehnten für einen gelebten UV-Schutz zur Prävention UV-bedingter Erkrankungen ein und arbeiten hierzu mit unterschiedlichen Partnern auf verschiedenen Ebenen zusammen. Aufgrund dessen und aufgrund der Bündelung dieser Anstrengungen im UV-Schutz-Bündnis mit entsprechenden Veröffentlichungen können seit Initiierung des Bündnisses etliche tief greifende Erfolge für den UV-Schutz und die Prävention UV-bedingter Erkrankungen verzeichnet werden. Hier sind unter anderem zu nennen:

- Im Jahr 2012 trat die Verordnung zum Schutz vor schädlichen Wirkungen künstlicher ultravioletter Strahlung (UV-Schutz-Verordnung) in Kraft [46]. Sie reguliert den Betrieb kosmetisch angewendeter UV-Bestrahlungsgeräte (Solarien) und beinhaltet unter anderem Vorschriften zu Bestrahlungsstärke, Fachpersonal und Informierung der Nutzer*innen.
- Im Jahr 2015 wurde die Richtlinie über die Früherkennung von Krankheiten bei Kindern (Kinder-Richtlinie) [47] um eine UV-Schutz-Beratung im Rahmen der U5-Untersuchung (6. bis 7. Lebensmonat) ergänzt.
- Seit 2015 sind das Plattenepithelkarzinom (helle Hautkrebsentität) und seine Vorstufen, die multiplen aktinischen Keratosen, als Berufskrankheit (§ 9 SGB [Sozialgesetzbuch] VII) anerkennungsfähig. Arbeitgeber*innen müssen in diesem Zuge die UV-Exposition ihrer Arbeitnehmer im Rahmen der Gefährdungsbeurteilung des Arbeitsplatzes berücksichtigen und für angemessenen UV-Schutz nach dem T‑O-P-Prinzip (technische Maßnahmen vor organisatorischen Maßnahmen vor persönlichen Schutzmaßnahmen) der Beschäftigten sorgen.
- Dermatologen können feststellen, dass das Bewusstsein für UV-Schutz in der Kindheit in den letzten Jahren gestärkt wurde. So wird beispielweise eine Abnahme der Anzahl UV-induzierter melanozytärer Nävi („Muttermale“), die als entscheidender Risikofaktor für die Entstehung des malignen Melanoms angesehen werden, bei Kindern und jungen Erwachsenen aufgrund von konsequentem Sonnenschutzverhalten verzeichnet (mündliche Mitteilung).
- Sowohl die Inhalte der konsentierten Empfehlung zu UV-Exposition und Vitamin D als auch des Grundsatzpapiers „Vorbeugung gesundheitlicher Schäden durch die Sonne – Verhältnisprävention in der Stadt und auf dem Land“ fließen in den letzten Jahren in Empfehlungen und Publikationen verschiedener Fachgremien ein, wie z. B. 2016 in die Empfehlung der Strahlenschutzkommission (SSK) zum „Schutz des Menschen vor den Gefahren solarer UV-Strahlung und UV-Strahlung in Solarien“ [5] und 2021 in die onkologische S3-Leitlinie „Prävention von Hautkrebs“ [40]. Etliche Partner des Bündnisses trugen mit ihrer fachlichen Expertise zur Aktualisierung der onkologischen S3-Leitlinie „Prävention von Hautkrebs“ bei, die unter anderem um das Thema Klimawandel und UV-Strahlung und um das Kapitel „UV-Exposition als mögliche exogene Schädigungsquelle für die Augen“ ergänzt wurde [40].
- Im Jahr 2021 gelang es, auf Basis des wissenschaftlichen Kenntnisstandes des UV-Schutz-Bündnisses die Klimawirkung „UV-bedingte Gesundheitsschäden“ in die Klimawirkungsrisikoanalyse 2021 (KWRA 2021) einzubringen [48]. Für diese Klimawirkung wurde, wie auch für die Klimawirkung „Hitzebelastung“, ein hohes Klimarisiko geschätzt und ein sehr dringendes Handlungserfordernis attestiert. Das Grundsatzpapier des UV-Schutz-Bündnisses wird im Hinblick auf die darin formulierte Maßnahme, dass ein Grundlagenkatalog zur Erstellung von Lehr- und Ausbildungsplänen für die Vermittlung von Fachkenntnissen über die gesundheitlichen Wirkungen der UV- und Hitzebelastung und den daraus ableitbaren Präventionsmaßnahmen zu erstellen ist, in dieser Analyse sowohl bei den Maßnahmen zur Klimawirkung „Hitze“ als auch zur neu betrachteten Klimawirkung „UV-bedingte Gesundheitsschädigungen“ zitiert.

## Schlussfolgerung

In den Jahren seit Gründung des UV-Schutz-Bündnisses hat sich die Arbeit für einen gelebten UV-Schutz und eine wirkungsvolle Prävention UV-bedingter Erkrankungen intensiviert. Die Aufgaben sind vielschichtig und bedingen nachhaltiges Handeln. Die interdisziplinäre Zusammensetzung des Bündnisses gewährleistet dafür eine umfassende und umfängliche sowie lösungsorientierte Herangehensweise zur Etablierung verhaltens- und verhältnispräventiver Maßnahmen und UV-Schutz-Verhalten in den Lebenswelten der Menschen auf Basis neuester wissenschaftlicher Erkenntnisse. Das Bündnis sieht seine Aufgaben darin, Impulse zur Schließung wissenschaftlicher Kenntnis- und Datenlücken zu geben. Des Weiteren wird es sein interdisziplinäres Wissen in Bezug auf Prävention, Diagnostik, Therapie und Patientenversorgung in nationale und europäische Gesundheitsprogramme einbringen, beispielsweise der Nationalen Dekade gegen Krebs des Bundesministeriums für Bildung und Forschung [15], der Nationalen Versorgungskonferenz Hautkrebs [18] und des Europäischen Krebsplans [49], sowie in die Normung. Darüber hinaus wird es die wissenschaftlichen Zusammenhänge und darauf aufbauende Aussagen aufbereiten und gegenüber allen verständlich kommunizieren.

Die interdisziplinäre Zusammensetzung des Bündnisses gewährleistet umfassendes und lösungsorientiertes Handeln

Ein Schwerpunkt der Arbeit des Bündnisses ist, die Prävention UV-bedingter Erkrankungen politisch zu verankern, beispielweise durch Einbringen des Ziels „Reduzierung der Mortalität und Morbidität UV-bedingter Erkrankungen“ in die etablierten nationalen Gesundheitsziele „Gesund aufwachsen“ und „Gesund älter werden“ – evtl. auch in das nationale Gesundheitsziel „Gesund rund um die Geburt“ im Rahmen des deutschen Präventionsgesetzes [50]. In Bezug auf UV-bedingte Hauterkrankungen stehen aufgrund des detaillierten wissenschaftlichen Kenntnisstandes die Zeichen dafür gut. In Bezug auf UV-bedingte Augenerkrankungen bedarf es dagegen weiterführender Forschung, z. B. hinsichtlich der wissenschaftlichen Aufarbeitung der Beteiligung von UV-Strahlung an der Entstehung degenerativer Netzhauterkrankungen wie der altersabhängigen Makuladegeneration.

## Fazit für die Praxis

- Auch heute ist der Schutz vor UV-Strahlung noch immer keine Selbstverständlichkeit.
- Das UV-Schutz-Bündnis setzt sich für gelebten UV-Schutz und eine wirkungsvolle Prävention UV-bedingter Erkrankungen ein.
- Die interdisziplinäre Zusammensetzung des Bündnisses gewährleistet eine umfassende und umfängliche sowie lösungsorientierte Herangehensweise zur Etablierung verhaltens- und verhältnispräventiver Maßnahmen und UV-Schutz-Verhalten in den Lebenswelten der Menschen auf Basis neuester wissenschaftlicher Erkenntnisse.

## Footnotes

1 UV-C-Strahlung ist nicht Bestandteil der natürlichen Sonnenstrahlung, sondern wird künstlich erzeugt und findet z. B. Anwendung zur Desinfektion von Luft, Wasser und Oberflächen.

2 Lebenswelten im Sinne des § 20 Absatz 4 Nr 2 PrävG und Sozialgesetz Buch V sind für die Gesundheit bedeutsame, abgrenzbare soziale Systeme insbesondere des Wohnens, des Lernens, des Studierens, der medizinischen und pflegerischen Versorgung sowie der Freizeitgestaltung einschließlich des Sports.

3 Hierzu zählt auch die Nutzung künstlich erzeugter UV-Strahlung in Solarien, die zu erheblichen gesundheitlichen Schädigungen führen kann. Eine Regulierung der Solariennutzung bzw. ein Verbot ihrer Nutzung ist daher auch als verhältnispräventive Maßnahme zu sehen.
